# Time and Causality: A Thermocontextual Perspective

**DOI:** 10.3390/e23121705

**Published:** 2021-12-20

**Authors:** Harrison Crecraft

**Affiliations:** GeoEx Analytics, Leesburg, VA 20176, USA; harrison@crecraft.net

**Keywords:** physical foundations, quantum mechanics, time, causality, entropy, entanglement, nonlocality

## Abstract

The thermocontextual interpretation (TCI) is an alternative to the existing interpretations of physical states and time. The prevailing interpretations are based on assumptions rooted in classical mechanics, the logical implications of which include determinism, time symmetry, and a paradox: determinism implies that effects follow causes and an arrow of causality, and this conflicts with time symmetry. The prevailing interpretations also fail to explain the empirical irreversibility of wavefunction collapse without invoking untestable and untenable metaphysical implications. They fail to reconcile nonlocality and relativistic causality without invoking superdeterminism or unexplained superluminal correlations. The TCI defines a system’s state with respect to its actual surroundings at a positive ambient temperature. It recognizes the existing physical interpretations as special cases which either define a state with respect to an absolute zero reference (classical and relativistic states) or with respect to an equilibrium reference (quantum states). Between these special case extremes is where thermodynamic irreversibility and randomness exist. The TCI distinguishes between a system’s internal time and the reference time of relativity and causality as measured by an external observer’s clock. It defines system time as a complex property of state spanning both reversible mechanical time and irreversible thermodynamic time. Additionally, it provides a physical explanation for nonlocality that is consistent with relativistic causality without hidden variables, superdeterminism, or “spooky action”.

## 1. Introduction

The nature of physical reality has been debated since the early twentieth century, when classical mechanics ceded its supremacy to quantum mechanics and relativity as fundamental descriptions of physics. Two of the most intractable conceptual problems facing physics are the problems of time and nonlocality.

### 1.1. The Problems of Time

Perhaps the most serious conceptual issue facing physics concerns the nature of time [1,2,3,4]. Physics describes change as fundamentally reversible and deterministic. Reversibility means that there is no fundamental distinction between past and future and that there is no fundamental arrow of time. Determinism means that the future is determined by the present. 

Determinism is a logical consequence of classical mechanics. Classical mechanics defines the microstate, which expresses everything that is measurable and knowable about a system, by perfect measurement in the absence of thermal noise. Perfect classical measurement reveals (in principle) the precise positions and motions of a system’s parts in addition to the forces acting on them. The application of Newton’s laws of mechanics to a precisely defined state completely determines all future states. 

Classical mechanics is deterministic, but it did not always embrace reversibility. Newton’s laws of mechanics are strictly empirical, and they accommodate friction as well as the irreversible dissipation of energy. Empirical observations of colliding clay lumps, for example, reveal the conservation of momentum but not of mechanical energy. Newton’s three laws of mechanics neither include nor imply the conservation of energy or reversibility. 

William Rowan Hamilton reformulated classical mechanics in the 1830s. He resolved a system into elementary particles, which have mass but no internal energy. With no internal energy a system’s total energy equals the sum of its particles’ kinetic and potential energies, and this defines the system’s mechanical and total energy. Hamiltonian mechanics interpreted heat as the mechanical energy of particles. Furthermore, it eliminated friction and the dissipation of mechanical energy into heat, and it formalized the conservation of energy into its conceptual model. Later, in a series of experiments in the 1840s, James Prescott Joule confirmed the equivalence of heat and mechanical energy, and in 1850 Rudolf Clausius wrote on the first law of thermodynamics, which formally established the conservation of energy. 

By interpreting heat as mechanical energy, Hamiltonian mechanics reinterpreted the first law of thermodynamics as the conservation of mechanical energy. Mechanical energy is quantified by its potential to do work, so its conservation means the conservation of work potential. The conservation of work potential, along with determinism, implies that we could, in principle, reverse the motions of a system’s particles and reverse its evolution without external work. This is the definition of thermodynamic reversibility and a logical consequence of the Hamiltonian conceptual framework (HCF). 

Boltzmann sought to reconcile the conflict between the reversibility of Hamiltonian mechanics and the thermodynamic arrow of time, as defined by the second law of thermodynamics and the production of entropy. Boltzmann described the entropy of a mechanical system by its disorder, which he defined by the number of accessible microstates consistent with its thermodynamic macrostate. The macrostate is a coarse-grained and incomplete description of the mechanical microstate, a consequence of thermal noise and imperfect measurement. He described the increase in entropy as the statistical tendency for large numbers of initially ordered particles to disperse and become increasingly disordered.

The dispersal of particles could be reversed in principle, resulting in a decrease in entropy without external work and without violating any fundamental laws of physics. Physics regards entropy as a measure of a macrostate’s imperfect description and an observer’s uncertainty of a system’s precise state, not as a fundamental property of physics. It likewise regards the second law of thermodynamics as a well-validated empirical principle, but not as a fundamental law of physics. 

With the discovery of quantum phenomena in the early twentieth century, it became clear that the laws of classical mechanics break down for very small particles and a new theory was needed. Quantum mechanics describes the quantum state via the Schrödinger wavefunction. The wavefunction expresses everything that is measurable and knowable about a system, and it therefore defines the quantum mechanical microstate. The wavefunction, similar to the Hamiltonian classical mechanical microstate, is both deterministic and reversible. 

The determinism and reversibility of the wavefunction are facts of its formulation. Individual quantum measurements and wavefunction collapse, however, are irreversible and random. Whether or not the underlying physical state is deterministic and reversible is a matter of interpretation and ongoing debate. Prevailing interpretations of quantum mechanics accept a key conclusion of the HCF: that the fundamental forces of physics are conservative. With conservative forces there is no dissipation, and this implies that, similar to its wavefunction description, an isolated quantum system’s physical state is both deterministic and thermodynamically reversible. 

Hamiltonian mechanics is both deterministic and thermodynamically reversible, but determinism implies another arrow of time: the arrow of causality. Determinism implies that causes have effects, and this defines a distinction between past and future as well as the irreversible arrow of causality. 

Reconciling the fundamental determinism and reversibility of the physical state with the empirical arrows of thermodynamic time and causality are two unresolved conceptual problems of time. 

### 1.2. The Problem of Nonlocality

Einstein, Podolsky, and Rosen (EPR) raised the issue of nonlocality in an article they published in 1935 [5]. If a pair of particles are emitted from a common source, they are then entangled by the virtue of sharing conserved properties, such as momentum or quantum spin. Quantum mechanics predicts that simultaneous measurements are correlated, such that measurements conserve the value of the conserved property that the pair inherited from their source. 

The EPR paradox is that the correlation of instantaneous and nonlocal measurements of physically separated particles seemingly violates relativistic causality, which prohibits the superluminal propagation of effects or information. One possible resolution would be the existence of additional “hidden” properties, locally inherited from the particles’ common source and not accounted for by quantum mechanics. These properties could carry information that determines the correlated measurement results, thereby eliminating the need for nonlocal “spooky action at a distance”. 

However, in 1964 John Bell published a statistical test for local hidden variables using randomly oriented detectors to measure quantum spin [6]. Numerous Bell test experiments have since demonstrated that spatially separated measurements do, in fact, statistically conserve quantum spin, and that the statistics of random measurements violate Bell’s test [7,8]. Experimental results and Bell’s theorem seem to prove that the correlation of spatially separated measurement results cannot be determined by local hidden properties [9]. 

There is a loophole in Bell’s proof of nonlocality, however. Bell’s theorem implicitly assumes that the settings for the randomly oriented Bell test measurements are, in fact, intrinsically random, meaning that they are uncorrelated. As Bell himself noted, it is possible to avoid the problem of superluminal speeds and spooky action if there is:

“…absolute determinism in the universe [and] the complete absence of free will. Suppose the world is super-deterministic, … the difficulty disappears. There is no need for a faster than light signal to tell particle A what measurement has been carried out on particle B, because the universe, including particle A, already “knows” what that measurement and its outcome will be”.

[10]

Superdeterminism [10,11] is simply the application of determinism to the universe as a whole. Superdeterminism implies that the entire history of the universe was determined at the beginning of time. It implies that the entangled particles and “random” experimental settings of Bell tests are, in fact, correlated as a consequence of their shared origin at the Big Bang. These correlations would violate the theorem’s assumptions and invalidate its conclusion. 

Superdeterminism cannot be empirically disproven; however, the idea that the universe could have started in such an extraordinary initial state to have determined the course of the universe’s evolution, including our own existence, thoughts, and choices, is so aesthetically distasteful that many physicists either ignore superdeterminism or reject it outright. The cost of rejecting superdeterminism, however, is steep. 

If we reject superdeterminism, Bell’s theorem and experimental data imply the coexistence of superluminal correlations and relativistic causality. There is no empirical conflict with this because it does not allow superluminal transmission of information, but there is also no explanation for how nonlocal measurements can be instantaneously correlated. This is the unresolved problem of nonlocality [5,6,7,8].

### 1.3. We Need a Better Conceptual Model

A conceptual model is an interpretation of physical reality. A good interpretation, first and foremost, must be able to reconcile the reversibility of a physical state with the empirical observations of irreversible transitions and the arrow of causality. It must reconcile determinism with the randomness of quantum measurements and wavefunction collapse. 

The Hamiltonian conceptual framework provides the foundation for classical mechanics, quantum mechanics, and relativity. The HCF’s unifying principle is that a physical state’s energy is completely defined in the limit of perfect measurement at absolute zero, in the absence of thermal noise. The logical implications of this are that (1) energy is resolved into kinetic energy and potential energy; (2) there is no dissipation; and (3) isolated physical states are therefore deterministic and thermodynamically reversible. 

Various HCF interpretations have been proposed to reconcile fundamental reversibility and determinism with empirical randomness and irreversibility. Implications of these interpretations include:

- The possibility of superposed live–dead cats (Copenhagen interpretation [12]);
- Exponentially splitting worlds (many-worlds interpretation [13]);
- Superdeterminism (classical statistical mechanics, relativity, and quantum hidden-variable theories [9]);
- Nonlocality (the nonlocal de Broglie–Bohm pilot wave theory [9]).

Not all interpretations of quantum mechanics adhere to the HCF’s assumption of objective reversibility. The consistent histories interpretation [14] defines the quantum state via perfect measurement with respect to an observer’s selected reference. Quantum Bayesianism [15] defines and updates the quantum state via information available to an observer. The von Neumann–Wigner interpretation attributes the physical collapse of the wavefunction to the consciousness of an observation event [16]. These non-HCF interpretations avoid the metaphysical implications of HCF interpretations, but they deny or ignore the objectivity of the physical state. 

The existing interpretations of the physical state either have untenable and untestable metaphysical implications or deny/ignore the objectivity of the physical state. We seek a third option. We seek an interpretation that (1) accepts the objective reality of physical states; (2) reconciles the determinism and thermodynamic reversibility of physical states with their irreversible and random transitions (including measurements); and (3) explains the superluminal correlation of measurements within the constraints of the subluminal arrow of relativistic causality. Satisfying these criteria requires a fundamental change in how we interpret time and causality. 

## 2. The Thermocontextual Interpretation of State

The thermocontextual interpretation (**TCI**) is an alternative to the existing HCF and subjective interpretations of physical reality. As with any conceptual model of physics, the TCI is an axiomatic system based on (1) empirically validated physical facts, (2) fundamental premises, and (3) a definition of perfect measurement. 

The TCI accepts the following as true, empirically validated facts:

1. Empirical conservation laws (e.g., energy, momentum, charges, and quantum spin);
2. Empirical laws of motion (e.g., Newton’s laws and quantum mechanics);
3. Empirical laws of interaction (e.g., law of gravitation and Planck’s law of radiation).

### 2.1. Postulates and Definitions of State

In addition to empirical facts and physical laws, the TCI’s interpretation of the physical state is based on the following postulates and definitions: 

Postulate one. The zeroth law of thermodynamics establishes that the temperature of a thermally equilibrated system is a measurable property. 

Postulate two. The third law of thermodynamics establishes that absolute zero can be approached but never be attained. 

Postulate three. There are no unobservable “hidden” variables. The physical properties of state are measurable and perfect measurement completely describes a system’s physical state.

**Definition** **1.** *A system’s ambient temperature, T_a_, equals the positive temperature of the system’s actual surroundings with which it interacts or potentially interacts.*

**Definition** **2.** *A system’s total energy, E, equals the system’s potential work as measured on the surroundings in the limit of absolute zero.*

**Definition** **3.** *A system’s exergy, X, is defined by its potential work as measured at the ambient surroundings.*

**Definition** **4.** *A system is in its ground state when its temperature equals the ambient temperature and its exergy equals zero. The system’s ground state is uniquely defined by its equilibrium with its ambient surroundings.*

**Definition** **5.** *A system’s ground state energy, Q_gs_, is the ambient ground state’s potential work capacity as measured at the limit of absolute zero.*

**Definition** **6.** *A system’s energy is defined by E_sys_ = E − Q_gs_.*

**Definition** **7.** *A system’s ambient heat is defined by Q = E_sys_ − X.*

**Definition** **8.** *Perfect measurement of a state is a reversible and open system process of transition from a system’s initial state to its ground state.*

**Definition** **9.** *A system’s entropy is defined by S = Q/T_a_.*

Postulate four (second law of thermodynamics). An irreversible process dissipates exergy into ambient heat. For irreversible change within an isolated system at constant *T_a_*, Δ*X* < 0. 

The TCI is a conceptual model and a simplification of reality. It is based on empirical facts and empirically justified assumptions that provide the logical foundation for the TCI and its explanations of empirical facts within its domain of empirical validation. 

Postulate one establishes temperature as a measurable property. The zeroth law of thermodynamics defines a system’s temperature by the measurable temperature of a thermometer or probe with which it is thermally equilibrated. 

Postulate two says that absolute zero can be approached but never attained. No system is perfectly isolated from its surroundings and all systems have a positive ambient temperature. Even the universe, which by definition has no physical surroundings, has an ambient energy background for the exchange of photons, defined by its cosmic microwave temperature at 2.7 kelvins. 

Postulate three is a statement about the TCI’s interpretation of physical reality. Postulate three defines physical reality by perfect measurement. The microstate, which expresses everything measurable and knowable about a system, is therefore a complete description of the physical state. “State”, without any qualification, will refer both to a system’s measurable microstate and to its underlying physical state.

Definition one defines a system’s ambient temperature by the temperature of the system’s ambient surroundings, whether or not the system is thermally equilibrated with its surroundings. Definition two follows the Hamiltonian conceptual framework by defining a system’s total energy with respect to absolute zero. However, whereas the HCF only considers total energy the TCI resolves total energy into thermocontextual components (definitions three and five–seven), given by:

*E* = *Q_gs_* + *E_sys_* = *Q_gs_* + *X* + *Q*. (1)

The system’s energy (*E_sys_*), exergy (*X*), and ambient heat (*Q*) are all thermocontextual properties of state, measured and defined with respect to the system’s ambient ground state (definition four), which defines their zero values. The ambient ground state energy (*Q_gs_*) is defined by measurements in the limit of absolute zero. 

The TCI’s ambient ground state energy is a generalization of quantum mechanics’ absolute ground state energy. Whereas the ambient ground state is in equilibrium with a system’s actual ambient surroundings at a positive temperature, quantum mechanics’ absolute ground state is defined at absolute zero. However, even in the limit of absolute zero, measurements reveal a positive ground state energy due to Heisenberg’s uncertainty principle and the observed randomness of a particle’s position and momentum.

The ambient temperature, not the system temperature, defines a system’s temperature of thermalization. If a system’s energy is fully thermalized at the ambient temperature, then it has no potential for work and it defines ambient heat.

If a system’s ambient temperature is lower than its temperature, then the system’s internal energy (heat) is only partially thermalized and it has positive thermal exergy. The thermal exergy and ambient heat contents of an increment of heat (*dq*) at temperature *T* > *T_a_* are empirically given by:

(2) $$dX_{q}=\left(\frac{T-T_{a}}{T}\right)dq\;\mathrm{and}\;dQ=\left(\frac{T_{a}}{T}\right)dq.$$

Thermal exergy is the maximum work that can be derived from heat and an ambient heat sink using a perfectly efficient heat engine. 

A system’s total exergy is the sum of its thermal exergy plus the nonthermal kinetic and potential energies of the system’s measurable components. A system’s measurable components generally are not elementary particles and can contain internal exergy (e.g., chemical or nuclear potential energy) in addition to the potential energy resulting from interactions with the particles’ external fields.

When combined with the law of conservation for energy, we can rewrite Equation (1) as:

(3) $$dE=dX+dQ+dQ_{gs}=0.\;$$

If the ambient surroundings are fixed then $dQ_{gs}$ equals zero, and Equation (3) shows that the dissipation of exergy is offset by an increase in ambient heat. Equation (3) also expresses the conservation of energy during changes in the ambient surroundings. A decline in the temperature of the ambient surroundings shifts ground state energy into system energy, and from Equation (2), this shifts ambient heat into exergy, but the isolated system’s total energy is conserved. 

**Figure 1:**
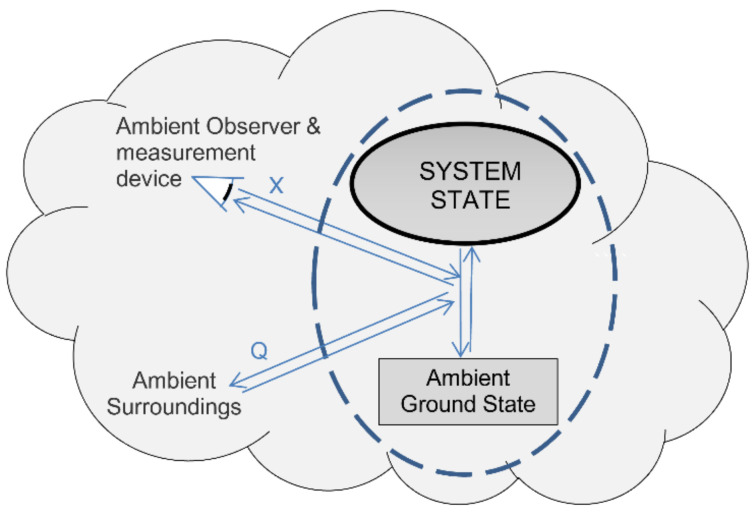
Perfect measurement is a reversible transformation from a system’s initial state to its ambient ground state reference. Perfect reversible measurement involves transfers of exergy (*X*) and ambient heat (*Q*) to the surroundings. Reversing the process restores the system’s initial pre-measurement state.

Definition eight defines perfect measurement of a system’s state as a reversible open system transition from an initial state into its ambient ground state. Before any interaction with its cooler ambient surroundings, a hot gas has positive exergy and ambient heat. In the limit of reversibility, we can cool the gas via a heat engine until it reaches its ambient ground state, and we can store the gas’s thermal exergy without dissipation or loss (Figure 1). The process thereby reversibly transitions the system into its ground state with zero system energy. Using the exergy stored in the surroundings, we can reverse the measurement process by reversibly pumping ambient heat back into the gas, restoring the gas to its original state.

Perfect measurement is defined as a reversible open system process, but reversible measurement is not always possible. The quantum watchdog effect (a continuous version of the Zeno effect) shows that a continuously measured (and measurable) state cannot change irreversibly [17]. The contrapositive of this is equally true: an irreversibly changing system is not continuously measurable. During radioactive decay, for example, exergy is irreversibly dissipated and the particle is not reversibly measurable. The particle therefore does not exist as a TCI state during transition. A system is reversibly measurable only *between* irreversible transitions, while it exists as a metastable state across an interval of time.

The TCI includes thermodynamic properties, but there is an important and fundamental difference between the thermocontextual state and the thermodynamic state. The TCI defines a state with respect to an ambient reference state that is fully thermalized at the system’s ambient temperature. Nonequilibrium thermodynamics, in contrast, assumes a non-isothermal system is thermalized at the system’s variable local temperature. There is no well-defined ambient temperature of thermalization, and it does not describe a state within the TCI. The thermodynamic description of an isothermal system, however, does describe the special case TCI state, in which the ambient temperature is defined by the system’s temperature. 

### 2.2. Entropy and Refinement

**Figure 2:**
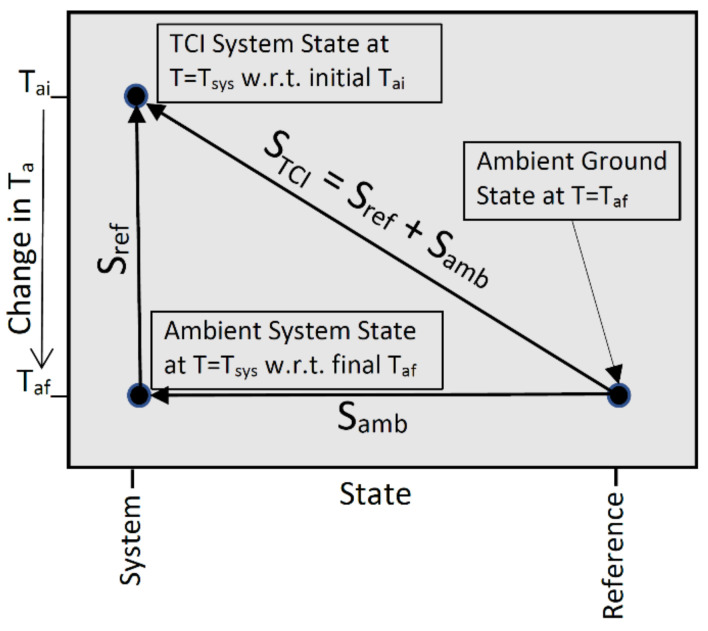
The two components of TCI entropy.

Definition nine defines entropy by *S* = *Q/T_a_*. As with ambient heat, the TCI entropy is a thermocontextual property of state that is defined relative to the system’s equilibrium ground state at the ambient temperature. The TCI resolves total entropy (diagonal vector in Figure 2) into two components: the ambient entropy (*S_amb_*, horizontal vector) and the entropy of refinement (*S_ref_*, vertical vector). 

Refinement was originally described by the consistent histories interpretation of quantum mechanics [14] as a consequence of a change in the measurement framework. The TCI extends this idea and defines the entropy of thermal refinement as a consequence of a decline in ambient temperature from an initial *T_ai_* to a final *T_af_*. The entropy of thermal refinement simply reflects a change in the ambient entropy due to a shift in the temperature scale for measuring entropy. If the ambient temperature is constant (*T_ai_* = *T_af_*) then *S_ref_* (vertical vector) equals zero and the ambient entropy and TCI entropy are equal.

The entropy of thermal refinement is defined by the integral:

(4) $$S_{ref}=\frac{1}{T_{af}}\int_{T_{af}}^{T_{ai}}dQ=\int_{T_{af}}^{T_{ai}}\frac{dq}{T}.\;$$

The differential *dQ* is the incremental change in the system’s ambient heat, thermalized at the fixed temperature *T_af_*, and *dq* is the incremental change in heat thermalized at temperature *T*. The first equality is based on definition nine and the second equality follows from Equation (2), with *T_ai_* = *T* and *T_af_* = *T_a_*. In both cases the changes are in response to the change in the ambient temperature prior to any heat transfer or other adjustments to the changed surroundings. 

For a system initially thermalized at its system temperature (*T_ai_* = *T_sys_*), as *T_af_* and the temperature of re-thermalization approach absolute zero, the ambient heat and the ambient entropy also approach zero. In the limit of *T_a_* = 0 kelvins the ambient entropy (horizontal vector) is zero. The TCI entropy therefore equals the entropy of refinement, and we get:

(5) $$S_{TCI}=S_{ref}=\int_{0}^{T_{sys}}\frac{dq}{T}\equiv S_{3^{rd}Law}.\left({\mathrm{for}\;T}_{a}=0\;K\right)\;$$

In the limit of an ambient temperature of absolute zero, *S_ref_* and *S_TCI_* are both equal to thermodynamics’ third law entropy, defined by the integral term. Conversely, the thermocontextual TCI entropy is a generalization of thermodynamics’ third law entropy, defined for the idealized special case of an ambient temperature of absolute zero. 

Refinement is about more than just a decline in ambient temperature: it is a consequence of any decline in a system’s equilibrium ground state energy. The conservation of energy means that a decline in a system’s ground state energy results in an increase in its system energy. Refinement also does more than just increase a system’s ambient heat and entropy: it also increases the system’s exergy. A system can be prepared in equilibrium with its environment of preparation with zero exergy and zero entropy, but when it is cast into lower-energy surroundings it will attain positive entropy and positive exergy.

### 2.3. Classical and Quantum States

A system’s TCI state is defined by its total energy with respect to absolute zero, by its ambient ground state, and by perfect measurement (definition eight, Figure 1). Table 1 describes the TCI interpretations of an ideal classical gas and a hydrogen atom. We initially consider the gas and hydrogen atom to be in equilibrium with their ambient surroundings. We assume that the gas is in equilibrium with a thermal bath at *T* = 500 K and fixed pressure and that the hydrogen atom is in equilibrium with a black body at *T* = 6000 K. This is below the hydrogen atom’s ionization temperature but hot enough that its single electron is distributed among multiple energy levels, and it is described by a superposed wavefunction.

The n-particle gas’s equilibrium state is defined by its equilibrium temperature and pressure (Table 1, rows one and two). The equilibrium gas is a special case TCI state with *T_a_* = *T*. The gas’s total energy is defined by the equilibrium temperature and pressure, and its thermocontextual properties all equal zero. The equilibrium gas defines the gas’s “e-contextual” state, where the TCI defines an e-contextual state by equilibrium with its ground state. 

The TCI generalizes the gas’s e-contextual state by defining the gas with respect to the system’s actual ambient surroundings. If we start with an equilibrium gas and lower its ambient temperature from 500 K to 300 K the e-contextual state is unchanged, but the gas’s thermocontextual state immediately changes, as shown in Table 1 (bottom five rows). In particular, prior to any interactions with the new surroundings at 300 K the gas at 500 K has a positive exergy and positive entropy of refinement with respect to its new ground state.

Quantum mechanics defines the hydrogen atom’s quantum state by its superposed wavefunction (Table 1, row one). The individual eigenfunctions are independent of temperature, but their complex weighting coefficients, and therefore the wavefunction, are functions of the hydrogen atom’s equilibrium temperature. The wavefunction and its equilibrium temperature uniquely specify the atom’s energy expectation value, defined in row two. The superposed wavefunction completely specifies the atom as it exists in equilibrium with its ambient surroundings in its e-contextual state.

If we perfectly isolate the hydrogen atom and lower its ambient temperature to 300 K its wavefunction and e-contextual state are unchanged. Its thermocontextual properties (bottom five rows of Table 1) do change, however. The thermocontextual state with respect to 300 K describes the atom with positive exergy and entropy. It is a generalization of its special case e-contextual microstate. 

If we lower the ambient temperature all the way to absolute zero, there is no possibility of dissipation, randomness, or irreversibility. The TCI describes a property or state that is defined with respect to absolute zero as a “z-contextual” property or state. Ambient heat and entropy are identically zero, and the system energy is equal to its exergy. The deterministic and reversible z-contextual state is an idealized special case of thermocontextuality, with ambient temperature equal to absolute zero. This applies to both classical and quantum states.

## 3. Thermocontextual Time

For millennia, time has been recorded by the sun’s shadow, the seasons, and, more recently, clocks. Lucia and Grisolia recently proposed a universal clock for measuring the irreversible passage of time based on irreversible thermodynamics of black-body radiation [18]. The TCI designates the irreversible changes in the surroundings, as measured by an external reference clock, as reference time. 

Newtonian mechanics described a second role of time: a mathematical parameter for equations of motion. The periodic motion of a harmonic oscillator, for example, is succinctly encapsulated by *x* = sin(*t*). The parameter “*t*” now has a dual role. As a measure of clock time it tracks the oscillator as it changes over reference time. As a parameter for an equation of motion it is an index of the oscillator’s position at any freely chosen instant. The TCI recognizes this dual role as two distinct times. Reference time is the time by which we record events and changes, as measured by an external reference clock. Mechanical system time is an index and property of state, divorced from the reference time of surroundings.

**Figure 3:**
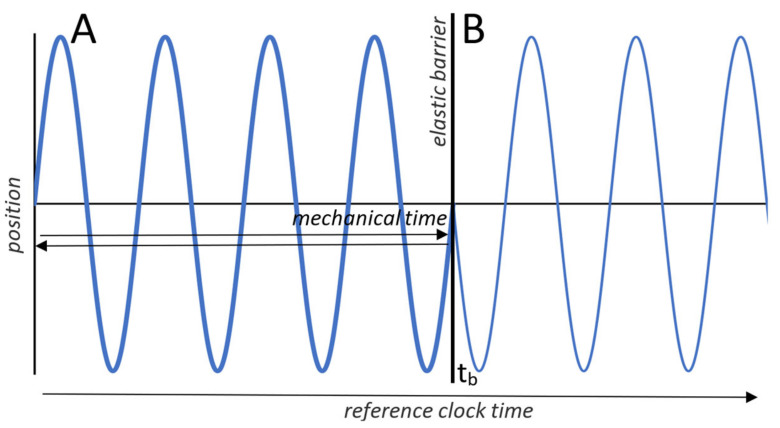
Mechanical and reference times. (**A**) Plot shows the trajectory of a one-dimensional harmonic oscillator over parallel advances in mechanical and reference times. (**B**) Mechanical and reference times decouple when the oscillator encounters an elastic barrier momentarily inserted at time t_b_. The oscillator and mechanical time, as an index of the oscillator’s position, reverse direction and retrace the trajectory over mechanical time. The trajectory in B shows the oscillator’s position across reference time, which continues to advance without bound.

For an undisturbed oscillator we can ignore the distinction between the two times by advancing mechanical time and reference time in parallel (Figure 3A). However, if an elastic barrier is inserted in the oscillator’s path, the two times decouple (Figure 3B). The oscillator and mechanical system time, as an index of its position, reverse direction, while reference time continues to advance unaffected. Mechanical system time, similar to the mechanical states that it indexes, is reversible and generally finite in extent, but reference time is irreversible and unbounded. 

Thermodynamics challenged the mechanical notion of system time as a reversible property of state. The second law of thermodynamics introduced the idea of a thermodynamic system time, which is contextually defined by a system’s process of entropy production and exergy dissipation. Thermodynamic system time can only increase with reference time; similar to reference time itself thermodynamic time is irreversible. 

The prevailing physical interpretations are noncontextual by implicitly assuming an ambient temperature of absolute zero. They consequently do not recognize irreversible dissipation or thermodynamic time. With absolute zero surroundings, reference time is reversible and there is no decoupling of reference and mechanical times. They view the irreversibility of thermodynamic system time and reference time as phenomenological manifestations of a single noncontextual time. The TCI, in contrast, is a thermocontextual interpretation of physical systems. It defines a system’s state with respect to the surroundings (or experimental setup) at positive temperature. The TCI recognizes contextual reference time, mechanical system time, and thermodynamic system time as three distinct and equally real components of time.

### 3.1. Thermodynamic Time

Thermodynamics defines the irreversible advance of system time for an isolated system by the production of thermodynamic entropy, *dS_TD_*, given by:

(6) $$dS_{TD}\equiv \frac{dq}{T}=\frac{dQ}{T_{a}}=-\frac{dX}{T_{a}}>0\;\left(\mathrm{for}\;\mathrm{isolated}\;\mathrm{system}\;\mathrm{at}\;\mathrm{fixed}\;T_{a}\right).\;$$

The identity relation defines the production of thermodynamic entropy, where *dq* is an increment of heat thermalized at temperature *T*. The first equality follows from definition nine and Equation (2) for fixed ambient temperature, and the last equality follows from (3). The inequality expresses the second law, which states that the thermodynamic entropy of an isolated system is constant for a reversible process and increases for any irreversible process. The second law of thermodynamics has been thoroughly validated by empirical observations.

The TCI formalizes the second law of thermodynamics according to postulate four in terms of the dissipation of exergy. The TCI does not define the second law by the production of thermodynamic entropy because it is not a thermocontextual property of state. Nor does the TCI define the second law by the production of TCI entropy, because it can include the entropy of refinement, which is reversible; if a change in ambient temperature is reversed, the change in the entropy of refinement is also reversed. The TCI establishes exergy as a physical property of state; it establishes the irreversible dissipation of exergy; and it establishes the thermodynamic arrow of time as a fundamental law.

The TCI defines thermodynamic time (*t_q_*) by:

(7) $$t_{q}=ln\left(\frac{X_{0}}{X\left(t_{ref}\right)}\right)t_{unit}.\;$$

*X*_0_ is the system’s initial exergy, *X* is the exergy at reference time *t_ref_* as measured by a reference clock in the ambient surroundings, and *t_unit_* is a chosen unit of reference time. The exergy and thermodynamic time for a first-order kinetic system, for example, are given by:

*X*(*t_ref_*) = *e^−^^λ^^tref^X*_0_ and *t_q_* = (*λt_unit_*)*t_ref_*, (8)

where *λ* is the constant of proportionality between dissipation rate and exergy. 

Equation (8) can describe the dissipation of exergy during radioactive decay. At time zero, the system’s exergy equals its initial exergy, *X*_0_, and as time advances toward infinity the system’s exergy approaches zero. As with exergy, thermodynamic time is a thermocontextual property of state. As a thermocontextual property, thermodynamic time is incompatible with and ignored by noncontextual interpretations of physics. 

### 3.2. Mechanical Time

Mechanical time is the time of classical mechanics, quantum mechanics, and spacetime. Mechanical time specifies a reversible system’s state at an arbitrary instant of mechanical time. It is defined as a parameter (*t*) in the mechanical laws of motion. In classical mechanics it is graphically represented by a coordinate on a system’s trajectory in phase space. In relativity it is graphically represented by a coordinate on the time axis of spacetime. Additionally, in quantum mechanics it is represented by a parameter of the time-dependent wavefunction. Mechanical time is a non-contextual property of state, independent of the surroundings. 

Mechanical time is conventionally defined as a real-valued coordinate, but this is merely a matter of convention. The TCI adopts a different convention by replacing (*i* × *t*), where *i* is the square root of negative one and *t* is real time, with the mathematically identical (*it_m_*), which we take as a coordinate of imaginary mechanical time. It is important to note that imaginary mechanical time does not involve a transformation of coordinates or a Wick rotation, it is simply a change in terminology and leaves all equations unchanged. For example, the TCI expresses the time-dependent quantum wavefunction for an isolated (fixed energy) and non-reactive quantum system by:

(9) $$\psi \left(x,it_{m}\right)=e^{\frac{-it_{m}E}{\hbar }}\psi_{o}\left(x\right).\;$$

Except for the change in the function’s time argument, (9) is identical to the conventional expression for the system’s time-dependent wavefunction. 

### 3.3. System Time and Reference Time

Equation (7) describes the continuous dissipation for a thermodynamic system. In the quantum limit, however, dissipation is discontinuous. A system’s positive exergy reflects its potential to transition irreversibly into its ground state, but the system can delay its reequilibration and can persist for a period of time as a metastable state. A particle of uranium 238, for example, can persist metastably for billions of years. As a metastable particle it does not dissipate exergy or change irreversibly. It is similar to any other stable particle—until it is not. At some point, a metastable particle irreversibly transitions into a new, more stable state of lower exergy, and this advances thermodynamic time. A metastable particle therefore requires both mechanical time and thermodynamic time to describe its behavior. 

**Figure 4:**
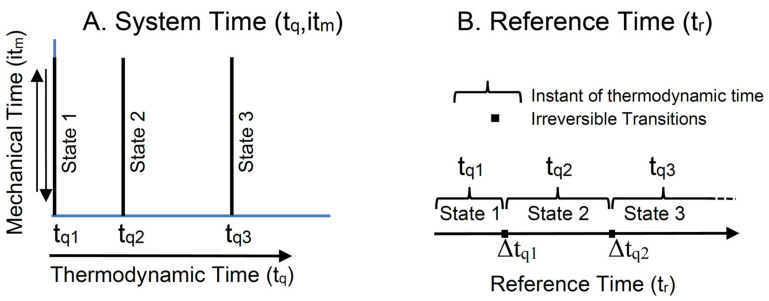
Complex system time and reference time. (**A**) Shows the complex system time plane, spanned by real-valued thermodynamic time (horizontal axis) and imaginary mechanical time (vertical axis). (**B**) Shows the irreversible advance in reference time over reversible intervals of system time at a fixed thermodynamic time, *t_qi_*, and between irreversible transitions over intervals of thermodynamic time, Δ*t_qi_*.

The TCI recognizes system time as a complex property of state, comprising both real-valued thermodynamic time and imaginary mechanical time. System time is represented by a point on the complex system time plane (Figure 4A). A change in mechanical time (vertical axis) conserves exergy, and it describes a reversible change in a state within a single instant of thermodynamic time, *t_qi_*. A change in thermodynamic time (horizontal axis) describes the irreversible dissipation of exergy and transition into a more stable state of lower exergy. 

System time deterministically specifies a system’s state at any particular point in complex time. The deterministic change in a system’s state with changes in system time defines a process. For an irreversible process, thermodynamic system time advances for each increment of exergy dissipation. A thermodynamic process therefore has a well-defined direction; this defines an arrow of thermodynamic causality by which we can distinguish cause from effect. A mechanical process, in contrast, is reversible. A mechanical process deterministically links two states, but determinism is time-symmetrical over mechanical system time and there is no distinction between cause and effect. 

Reference time (t_r_ in Figure 4B) is the time of relativity as measured by an external clock. It is the time by which we measure the advance of a light cone; it defines the arrow of relativistic causality. Effects always follow causes in reference time, even when they are connected over system time by time-symmetrical determinism. Reference time provides the time scale across which a system’s events are observed and velocities are measured. As with an observer’s clock, it ticks forward whether the system’s time proceeds reversibly or irreversibly. 

The TCI recognizes system time as distinct from irreversible reference time. Reference time flows irreversibly toward the future, independent of a system’s state and without any clear bound. System time is a property of state, specifying a system’s state at any selected instant of system time. The real thermodynamic component of system time increases irreversibly with reference time, and as with reference time it irreversibly increases without bound. The imaginary mechanical component of system time, in contrast, indexes the reversible changes in mechanical states. The Poincaré recurrence theorem [19] shows that reversible states cycle over time. This means that mechanical time, as an index of reversible mechanical states, is confined to a finite interval.

By resolving time into its three components, the TCI distinguishes between time-symmetrical determinism and the arrow of causality, and it recognizes the thermodynamic arrow of time as fundamental. 

## 4. Instantiation and Actualization 

Definition eight defines perfect measurement as a reversible process of transition into a system’s ground state (Figure 1). The transition involves the transfer of ambient heat to the ambient surroundings and the transfer of exergy to an external system, which reversibly records the measurement result. To gain a deeper understanding of measurement and of transitions more generally, we need to separate the transition process into two separate subprocesses of instantiation and actualization. These are given by definitions 8.1 and 8.2:

**Definition** **8.1.** *Instantiation is the reversible transition of an indefinite positive-entropy microstate to a definite zero-entropy microstate. Instantiation transfers the system’s ambient heat and entropy to the surroundings while preserving its mass and exergy.*

**Definition** **8.2.** *Actualization involves the reversible transition of an instantiated zero-entropy and positive-exergy microstate to a more stable lower-exergy state. Actualization is the work on an external system during the transfer of exergy from the system to the surroundings.*

The definitions for instantiation and actualization apply to any nonequilibrium system, quantum or classical, as it approaches equilibrium with its surroundings. Before we can discuss quantum instantiation and wavefunction collapse, however, we first need a statistical formulation of TCI entropy. 

### 4.1. Statistical Entropy

The statistical mechanical interpretation of thermodynamics’ third law entropy (5) is Gibbs entropy, defined by:

(10) $$S_{Gibbs}\equiv -k_{B}\underset{i}{\sum }p_{i}\ln\left(p_{i}\right)=S_{3^{rd}Law}.\;$$

Gibbs entropy generalizes Boltzmann entropy by assigning a probability *p_i_* to each classical mechanical microstate, “*i*”. Each microstate is precisely defined by perfect measurement at absolute zero. Each *p_i_* expresses the subjective probability that a system exists in microstate “*i*”, and Gibbs entropy expresses an observer’s subjective uncertainty of a system’s actual microstate. Gibbs entropy is an informational entropy. Except for a constant multiple, it is identical to Shannon’s information entropy.

Except for the constant, *k_B_*, Equation (10) also defines von Neumann entropy [20]. The summation for the von Neumann entropy of a mixed quantum state is over the individual component states’ wavefunctions, and the *p_i_* are their respective weightings. It follows that a “pure” quantum state, having a single wavefunction, has zero von Neumann entropy. As with Gibbs entropy, von Neumann entropy expresses an increase in an observer’s subjective uncertainty of a system’s actual state following wavefunction collapse of a pure state into a mixed state. Gibbs and von Neumann entropies are information entropies; they are not physical properties of state.

Equation (5) showed that third law entropy is a special case of contextual TCI entropy, with ambient temperature equal to absolute zero. Equation (10) shows that Gibbs/von Neumann entropy is also a special case of TCI entropy. Conversely, TCI entropy generalizes Gibbs/von Neumann entropy for a system as it exists with respect to its actual surroundings. We can therefore express the generalized contextual TCI entropy statistically, by:

(11) $$S_{TCI}=-k_{B}\underset{i}{\sum }P_{i}\ln\left(P_{i}\right).\;$$

The summation in (11) is over measurable microstate potentialities. Multiple potentialities and positive entropy are created by refinement and the change in surroundings to a lower ground state energy. For a classical system, microstate potentialities are a contextual generalization of microcanonical microstates defined for a positive ambient temperature. For a quantum system the observable microstate potentialities and entropy are defined by its Hilbert space basis, which reflects the system’s contextual surroundings or experimental setup. The refinement of a quantum system results from a change in its Hilbert space basis [14].

The *P_i_* in (11) are the objective probabilities that microstate potentiality “*i*” will be randomly selected when the system’s entropy is reset to zero, at which point the system reverts to a single measurable potentiality. A classical system’s entropy is reset to zero when the system’s entropy is entirely transferred to the new surroundings. A quantum system’s entropy is reset to zero when its superposed state vector is projected onto one of its new basis vectors. This describes the random collapse of an indefinite positive-entropy superposed state to a definite zero-entropy state. The probabilities in (11) are independent of observation or an observer’s knowledge, and the TCI entropy is objectively defined. 

### 4.2. Instantiation and Wavefunction Collapse

**Figure 5:**
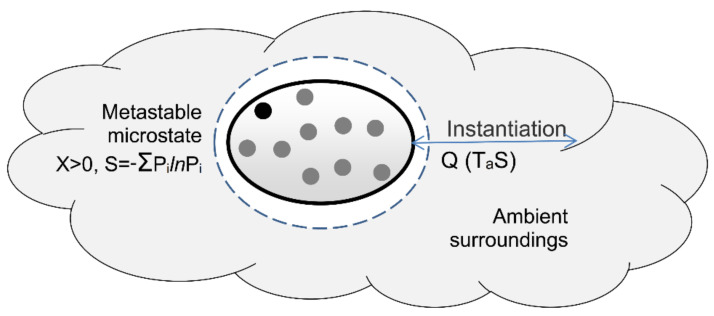
Instantiation of metastable microstate. The microstate initially has positive entropy and comprises multiple measurable potentialities (dots). Instantiation involves the transfer of ambient heat and entropy to the surroundings. This reduces the microstate’s entropy to zero and randomly instantiates a single zero-entropy potentiality (black dot).

Figure 5 illustrates the instantiation of a microstate potentiality. The positive-entropy microstate contains multiple microstate potentialities, represented by the dots; its positive entropy is given by (11). Instantiation reversibly exports ambient heat and entropy to the surroundings in a process of derandomization. Derandomization resets the system’s entropy to zero and selects a single microstate potentiality (Figure 5). The process thereby randomly and reversibly instantiates a zero-entropy microstate potentiality from the metastable microstate’s multiple potentialities. 

We can illustrate the refinement and instantiation of a quantum system by considering an entangled pair of atoms sharing quantum spin, a conserved property. The Stern–Gerlach experiment [21] first established spin as a measurable and quantized property of state. The Stern–Gerlach experiment sent a horizontal beam of silver atoms through a magnetic field. Silver has one unpaired electron, and its spin creates a magnetic dipole. When the magnetic field is applied it exerts either an upward or downward force on the atoms depending on the orientation of the dipole. Observations reveal two discrete diverging trajectories, indicating that the quantum spins are quantized, described as either “spin-up” or “spin-down”.

The magnetic field defines the atoms’ ambient surroundings. Before an atom interacts with the magnetic field its spin state cannot be measured, and, according to postulate three, it therefore has no spin. It only has spin potentialities, contextually defined by its experimental setup. With two measurable spin potentialities, the atom is described as a superposed state, and, according to Equation (11), it has positive entropy. 

Interaction of the atom with the magnetic field causes its superposed wavefunction to collapse randomly into a definite observable potentiality, diverted either upward or downward. As with classical instantiation, wavefunction collapse involves the transfer of entropy to the surroundings, derandomization, and the random instantiation of a definite measurable zero-entropy potentiality.

Random instantiation reveals an apparent connection between mechanical time and temperature within the noncontextual framework of statistical mechanics. Statistical mechanics treats virtual potentialities as actual microstates indexed over mechanical time. It defines the probability of observing a particular microstate by the fraction of mechanical system time indexed to that microstate. The probability of observing that microstate is also related to inverse temperature by Boltzmann’s partition function. The partition function is proportional to exp(−*E*/*k_B_T_a_*), where k_B_ is the Boltzmann constant and *T_a_* is the temperature of thermalization. Equating the two probabilities implies a connection between ambient temperature and time. The TCI, however, does not recognize a connection between ambient temperature and mechanical time because microstate potentialities are intrinsically random, which means that they cannot be indexed as a function of time.

### 4.3. Actualization and Measurement

As described in the previous section, the first stage of measurement is the instantiation of one of a system’s microstate potentialities. This involves the transfer of entropy and randomness to the surroundings in addition to the random instantiation of a definite zero-entropy microstate (Figure 5). 

**Figure 6:**
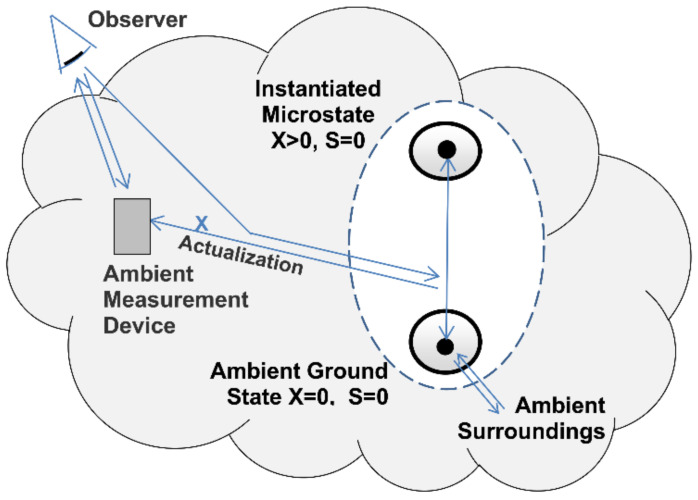
Perfect measurement. Perfect measurement is a reversible open system transition from an instantiated zero-entropy microstate to its ambient ground state reference. During perfect measurement the initial state’s exergy actualizes a record of the transition on the surroundings.

The second stage of measurement is actualization (Figure 6). Actualization is the transition from an instantiated positive-exergy and zero-entropy microstate to the ground state. The transition is reversible and adiabatic. It’s being reversible means that there is no dissipation of exergy or production of entropy within the system. It’s being adiabatic means the process is isolated to the exchange of heat and entropy. The initial state’s exergy is reversibly transferred to the surroundings where it can do the work of actualizing a measurement result. 

The actualization of a high-energy photon as it transitions into its ambient ground state, for example, could involve the work of actualizing a photochemical reaction and recording the photon’s point of impact on a photographic film. Actualization is more than just recording a measurement result, however. It more generally describes the work done on a system’s surroundings as an instantiated microstate transitions into a more stable state of lower exergy.

The actualization of a measurement result or other interaction with the surroundings is generally irreversible, but irreversibility is confined to the system’s surroundings. Changes in the system itself are reversible (Figure 6). By restoring the exergy transferred to the surroundings the system’s pre-measurement zero-entropy microstate is restored and the process is reversed.

## 5. Entanglement and Nonlocality

As described in Section 1.2, numerous Bell test experiments have shown that measurements on entangled systems are correlated, even if measurements are simultaneous and spatially separated. Before addressing how simultaneous measurements can be reconciled with relativistic causality, we first need to address the meaning of entanglement. The TCI defines entanglement as:

Definition ten. Physically separated particles are entangled if they have properties linked by a deterministic and thermodynamically reversible connection.

Determinism simply means that a change in one particle correlates to a specific change in the other particle. Thermodynamic reversibility means that there is no dissipation.

### 5.1. Entanglement—A Mechanical Illustration

This section illustrates entanglement and the distinction between causality and time-symmetrical determinism. We start with a simple classical system and then proceed to photons. We first consider frictionless gears linked together in an open circular chain so that an observer, Alice, can simultaneously observe both terminal gears. Any disturbance of one terminal gear is transmitted to the other terminal gear. As per definition ten, the terminal gears are entangled by a deterministic and thermodynamically reversible connection. 

The deterministic link connecting the entangled gears is frictionless and thermodynamically reversible, but it is still subject to irreversible relativistic causality. The physical interaction involves electrostatic interactions between the atoms, and the propagation of effects cannot exceed the finite speed of light as measured across reference time. If Alice rotates gear A she will see gear B respond a small time later. If she rotates gear B she will see a response in gear A after a short delay. Alice observes a clear distinction between cause and effect over her reference time. This illustrates the irreversible arrow of relativistic causality.

When we consider what happens over system time we have the same deterministic and thermodynamically reversible chain of events, but the distinction between cause and effect disappears. The propagation of effects for the thermodynamically reversible chain of gears is symmetrical across system time. A disturbance of A is transmitted across system time and affects B, but with time symmetry we can just as easily say that the disturbance at B affects A. This expresses the idea of time-symmetrical determinism. Time-symmetrical determinism occurs within an instant of thermodynamic time and is independent of reference time as well as the arrow of relativistic causality. 

Classical gears have zero entropy and a definite state. A quantum analogue would be a zero-entropy pair of entangled photons. A vertically polarized photon can be down-converted into a zero-entropy pair of entangled photons with the same vertical polarization. If the photons encounter vertically polarized analyzers, the measurement of one photon is deterministically linked to the other’s measurement by their electromagnetic oscillations, reversibly transmitted across system time and linked by their common source. As with the entangled gears, the entangled photon pair’s contextual state has a single measurable potentiality and zero entropy, and their measurements are deterministically linked. 

Photons are not classical particles, however. This becomes clear when we set the polarized analyzers oblique to the entangled pair’s vertical polarization. The photon pair then becomes superposed with multiple measurable potentialities, and from (11), it has positive entropy. Bell’s theorem and experimental results prove that the statistics of measurement are not merely informational, based on uncertainties or unknown hidden parameters. They prove that measurement results are objectively random and that its positive entropy is an objective property of the physical state. There is no classical analogue to this situation because classical entropy is strictly informational. The physical entropy of superposed quantum states, not entanglement, is the key distinction between classical and quantum mechanics.

### 5.2. Quantum Nonlocality—Just the Facts

**Figure 7:**
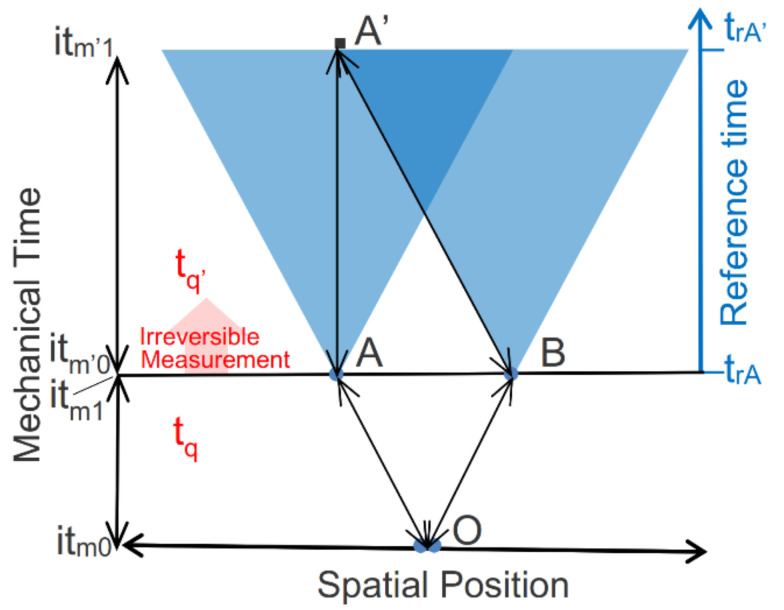
Instantaneous correlation of spatially separated measurements. The Figure spans space (projected onto the horizontal axis) and mechanical time (left axis). Mechanical time spans two reversible intervals at two distinct instants of thermodynamic time, separated by the irreversible actualization of photon measurement results. Superimposed on the diagram are light cones advancing across the reference time for the measurement events at A and B (right axis). Each light cone shows the domain of causality from A or B within the constraints of relativity.

Quantum nonlocality has been documented by numerous Bell-type experiments. A typical experiment involves a source which emits a pair of photons entangled by polarization. For simplicity, we consider the interaction of the photon pair with vertically oriented polarized analyzers at points A and B (Figure 7). The photons are emitted from their source at point O in opposite directions (horizontal axis). After an interval of time (vertical axis), Alice and Bob simultaneously measure their photons’ polarizations at A and B, at which point the photons’ measurement results are recorded. 

If the photons are entangled in parallel polarization, but they are not initially plane-polarized, then Alice and Bob each have a 50% probability of measuring a vertically or horizontally polarized photon regardless of their analyzer’s orientations. Because of the pair’s entanglement, however, the measurements are strictly correlated: if Bob measures a vertically polarized photon, Alice also measures a vertically polarized photon. If Alice measures a horizontally polarized photon Bob also measures a horizontally polarized photon. The instantaneous correlation of physically separated measurements at points A and B outside of each other’s light cone of relativistic causality (Figure 7) graphically illustrates the nonlocality of these measurement results.

### 5.3. Nonlocality and Bell Locality—An Explanation

Numerous Bell test experiments have shown that the statistics of correlated measurements violate the Bell theorem’s assumptions of local realism. Locality, as used by Bell, means that effects cannot propagate superluminally (relativistic causality). Realism means that properties have well-defined values even when not measured. Most interpretations of quantum mechanics, including the TCI, accept relativistic causality (Bell locality) and reject realism. Bell’s theorem, experimental results, and the rejection of realism and superdeterminism imply the coexistence of Bell locality and nonlocal correlations of spatially separated measurements. 

Any viable interpretation must be consistent with relativity. The TCI, however, goes beyond consistency by providing a viable explanation for the coexistence of nonlocality and Bell locality. This section provides an explanation of how spatially separated photons can instantly coordinate their measurement responses within the constraints of relativist causality. 

The photons are initially created at their source as a definite zero-entropy entangled pair with no measurable polarization. Once the photons are emitted, however, they are refined by the polarizers, which define a new and changed reference. Refinement results in two measurable microstate potentialities, parallel or perpendicular to the polarizer, and by (11) a positive entropy. The superposed and entangled photons follow reversible and deterministic trajectories from their initial entangled state at point O to their points of measurements at A and B, illustrated by A↔O↔B (Figure 7). 

When the superposed and entangled pair encounter a vertically oriented filter at point A or B and time (t_q_,it_m1_) (Figure 7) the interaction transfers entropy to the surroundings and derandomizes the photon pair. This resets the entangled pair’s entropy to zero and instantiates one of their potentialities as a definite zero-entropy microstate (see Figure 5). Entanglement by parallel polarization constrains the pair’s instantiated and entangled microstate to either $\left(↕↕\right)$ or $\left(↔↔\right)$. All of this deterministically occurs over an interval of time-symmetrical mechanical time, *it*_*m*0_–*it*_*m*1_, within an instant of thermodynamic time at *t_q_* (Figure 7). 

The transition of the instantiated photon pair into its ground state then actualizes measurement results on detectors at A and B. Dissipation during the measurement process advances the thermodynamic system time from *t_q_* to *t_q’_* and sets mechanical time to a new interval of time symmetry, *it*_*m*’0_–*it*_*m*’1_. 

The light cones at A and B show the domains of relativistic causality from each measurement event. Alice and Bob reversibly record the actualized measurement results for their photons at points A and B at system time (*t_q’_*,*it*_*m*’0_) and Bob transmits his result to Alice via a light signal. Alice receives the results recorded by Bob at point A’ and time (*t_q’_*,*it*_*m*’1_) and is able to verify that Bob’s results are correlated with hers. Alice’s and Bob’s observations of their measurement results, Bob’s transmission of his results, and Alice’s recording of his results are conducted across time-symmetrical mechanical time within the instant of thermodynamic time *t_q’_*.

Whereas Alice and Bob reversibly record their correlated measurement results within a single instant of thermodynamic time, their record of events over reference time is very different. The righthand axis of Figure 7 shows the record of Alice’s measurement events at points A and A’, as measured by her reference clock. Alice’s measurement at her reference time, *t_rA_*, and her recording of Bob’s measurement result at *t_rA’_* are reversibly linked within an instant of thermodynamic time via A↔A’↔B (Figure 7), but her reference time ticks irreversibly forward. Even though Bob and Alice cannot receive the other’s results until after they conduct their own measurements, they each conclude the other’s measurement was conducted simultaneously with their own and that the measurement results were instantaneously correlated. 

The TCI reconciles the instantaneous correlation of spatially separated measurements (nonlocality) and relativistic causality (Bell locality). Relativistic causality and observations are defined across irreversible reference time, as measured by the continuous advance of an inertial observer’s reference clock. Nonlocal measurements are deterministically correlated across time-symmetrical mechanical system time within a single instant of thermodynamic time. By distinguishing between complex system time and reference time, the TCI explains how the simultaneous correlation of nonlocal measurements can compatibly coexist with relativistic causality without spooky action, hidden variables, or superdeterminism.

## 6. Summary and Conclusions

The thermocontextual interpretation of physical reality starts with the same empirical facts as those of the existing interpretations, but it differs in the assumptions by which it interprets those facts. The TCI is strictly based on empirically justifiable assumptions. It contextually defines a state with respect to a system’s actual surroundings at a positive ambient temperature and with respect to an external reference time. It defines entropy, exergy, ambient heat, and reference time as measurable thermocontextual properties of state. It establishes the irreversible dissipation of exergy as a measure of fundamental irreversibility and a measure of irreversible thermodynamic system time. It defines mechanical system time as the conventional time of mechanics, described as a coordinate of spacetime or coordinate of the time-dependent wavefunction. 

The existing conceptual interpretations of physical reality can be divided into three distinct types: z-contextual, e-contextual (Section 2.3), and subjective interpretations (Section 1.3). Subjective interpretations include quantum Bayesianism [15], the Wigner–von Neumann interpretation [16], and the consistent histories interpretation [14]. They either deny or ignore an objective physical reality. Subjective interpretations include the observer or observer’s choices as essential elements of the quantum state’s definition and transitions. 

Z-contextual interpretations describe the physical state in terms of precise and definite properties defined in the absence of thermal noise, the implications of which are a noise-free ambient reference at absolute zero, no dissipation, and superdeterminism. Superdeterminism, in turn, implies an extraordinarily improbable initial state of the universe that has dictated in complete detail its past, present, and future. The future, as well as the past, is set in stone. Z-contextual interpretations include classical mechanics, relativity’s block model of the universe [22], and hidden-variable theories of quantum mechanics. 

E-contextual interpretations assume that the wavefunction is a complete description of an isolated system’s physical state, the implication of which is that isolated systems are likewise reversible functions of time and therefore at equilibrium. At equilibrium nothing actually happens so determinism and causality are not even applicable to e-contextual interpretations. E-contextual interpretations include the Copenhagen interpretation, the many-worlds interpretation, and quantum thermodynamics [23]. 

Z-contextual and e-contextual interpretations define two idealized special cases of the TCI. Z-contextuality defines the state with respect to absolute zero, which allows information-preserving transformations from one reference state to another. E-contextuality defines a system’s state with respect to its unique equilibrium reference, for which the wavefunction is a complete description (Section 2.3). In both cases, states are time-symmetrical and independent of their actual surroundings. 

The TCI bridges these idealized extremes. It describes a system as it exists as a non-equilibrium state with positive entropy and exergy, defined with respect to its positive-temperature ambient surroundings. This is the zone of thermocontextuality, and this is where irreversibility and randomness occur. 

The TCI distinguishes between system time and reference time. Reference time is the time of relativity, with respect to which the speed of light and the light cone of relativistic causality are defined. Reference time and the irreversible arrow of causality are both defined with respect to an observer’s clock. As with the observer’s clock, reference time irreversibly flows toward the future.

The TCI defines system time as a complex property of state spanning both reversible mechanical time and irreversible thermodynamic time. Thermodynamic time describes the irreversible dissipation of exergy associated with the transition of metastable states to states of lower exergy and higher stability. Mechanical time describes the reversible changes for equilibrium states and for metastable states between irreversible transitions. An equilibrium or metastable state has no dissipation, it exists within an instant of thermodynamic time, and it is reversible over mechanical system time. Whereas thermodynamic time can only increase over reference time, mechanical time can proceed forward or backward over reference time. 

An interpretation is “good” if it is consistent with empirical observations; precise; parsimonious in its assumptions; explanatorily broad; falsifiable; and promotes scientific progress [24]. The TCI is consistent with observations and it respects empiricism by rejecting z-contextuality as an unattainable idealization contrary to empirical facts. It is parsimonious by rejecting hidden variables, which cannot be empirically validated. The TCI explanations are falsifiable, but the TCI’s implications and explanations are validated by empirical observations. 

The TCI’s explanations are broad. It explains the thermodynamic arrow of time and the measurement problem without invoking empirically consistent but untestable and untenable metaphysical implications. It reconciles the irreversible arrow of causality and the time symmetry of states. Additionally, it explains the coexistence of nonlocality and relativity in terms of fundamental principles, without hidden variables, spooky action, or superdeterminism. The TCI can account for the cosmological arrow of time, which is related to the universe’s expansion and declining ambient temperature [3]. A declining ambient temperature creates a source of new exergy from a declining ground state energy. The cosmological arrow of time ensures that heat death, defined by an equilibrium state of zero exergy, will never occur as long as the universe continues to expand, its ambient temperature continues to cool, and new exergy is created.

The TCI also suggests new avenues of scientific progress. It extends the scope of physics from its traditional focus on states to irreversible dissipative processes. It thereby opens up new avenues for the investigation of self-organizing dissipative structures. Dissipative structures spontaneously develop in systems that are open to external sources of exergy [25]. They underly the evolution of far-from-equilibrium systems in physics, from quantum to astronomic scales; in biology, from molecular to ecological scales; and in economic and social systems [26].

As with any model the TCI is a simplification of reality, but it is an important step forward. By all measures, the TCI is a good interpretation of physical reality. 

## Figures and Tables

**Table 1:** TCI states and thermocontextual properties.

Energy Component	n-Particle Ideal Gas	Hydrogen Atom at Temperature T	Thermocontextual?
State Description	Equilibrium state (T,P; V = nk_B_T/P)	$\Psi \left(T\right)=\underset{i}{\sum }c_{i}\left(T\right)\psi_{i}\;\;\mathrm{where}\;\;\underset{i}{\sum }{\left|c_{i}\left(T\right)\right|}^{2}=1$	No
Energy (total)	E = nk_B_T	$\langle E\left(T\right)\rangle =\underset{i}{\sum }E_{i}\times {\left|c_{i}\left(T\right)\right|}^{2}=\underset{j}{\sum }p_{j}\left(T\right)E_{j}{}^{\left(1\right)}$	No
Q_gs_ (ground state energy)	Q_gs_ = nk_B_T_a_	Q_gs_ = $\langle E\left(T_{a}\right)\rangle =\sum_{j}p_{j}\left(T_{a}\right)E_{j}$	Yes
E_sys_ (system energy)	E_sys_ = E − Q_gs_	$\langle E_{\mathrm{sys}}\left(T\right)\rangle =\langle E\left(T\right)\rangle -Q_{\mathrm{gs}}=\underset{j}{\sum }E_{j}\left(p_{j}\left(T\right)-p_{j}\left(T_{a}\right)\right)$	Yes
Q = T_a_S_TCI_ = T_a_S_ref_^(2)^ (ambient heat)	$Q=T_{a}\left(\int_{T_{a}}^{T_{\mathrm{sys}}}C_{v}\left(T\right)\frac{dT}{T}\right)$	$\langle Q\left(T\right)\rangle =T_{a}\left(\int_{T_{a}}^{T}\left(\frac{\partial E\left(T\right)}{\partial T}\right)\frac{dT}{T}\right)$	Yes
X (exergy)	X = E_sys_−Q	$\langle X\left(T\right)\rangle =\langle E_{\mathrm{sys}}\left(T\right)\rangle -\langle Q\left(T\right)\rangle$	Yes
S_TCI_ = Q/T_a_ (entropy)	$S_{\mathrm{TCM}}=\frac{Q}{T_{a}}=\int_{T_{a}}^{T_{\mathrm{sys}}}C_{v}\frac{dT}{T}$	$\langle S_{\mathrm{TCM}}\left(T\right)\rangle =\frac{Q\left(T\right)}{T_{a}}=\int_{T_{a}}^{T}\left(\frac{\partial E_{\mathrm{sys}}\left(T\right)}{\partial T}\right)\frac{dT}{T}$	Yes

k_B_ = Boltzmann constant. C_v_ = volumetric heat capacity. Angle brackets indicate the expectation (and time-averaged) values. (1) The expectation energy value is defined by a weighted sum over the eigenfunction energies, E_i_, which typically includes degenerate and unresolvable eigenfunctions sharing the same energy. The TCI expresses the energy expectation value as a weighted sum over its discrete measurable microstate energies, E_j_, and its probabilities, p_j_. (2) The gas and hydrogen atom are related to their ambient states by a change in ambient temperature only, prior to any other changes. S_TCI_ therefore equals S_ref_ (vertical vector in Figure 2).

## Data Availability

Not applicable.
